# Assessment of Radiological Outcomes of Medial Meniscus Posterior Root Tears Associated With Meniscal Extrusions After Open Wedge High Tibial Osteotomy

**DOI:** 10.7759/cureus.46884

**Published:** 2023-10-12

**Authors:** Yavuz Selim Karatekin, Harun Altinayak

**Affiliations:** 1 Department of Orthopedics and Traumatology, Samsun Training and Research Hospital, Samsun, TUR

**Keywords:** meniscal extrusion, meniscus tear, varus deformity, medial meniscus posterior root tear, tibial osteotomy, open wedge osteotomy

## Abstract

Objective

The aim of this study is to compare preoperative and postoperative radiological results in knees with medial meniscus posterior root tears (MMPRT) and varus alignment, with a particular emphasis on medial meniscal extrusion (MME), following high tibial osteotomy (HTO) without root repair.

Method

Patients who underwent open wedge HTO for medial compartment osteoarthritis between January 2015 and December 2020 were retrospectively reviewed. The inclusion criteria were defined as patients with preoperative and postoperative magnetic resonance imaging (MRI) and weight-bearing radiographs including radiological images of the entire lower extremity. After conducting data screenings, patients diagnosed with a preoperative MMPRT were included in the study. Patients underwent measurements of medial proximal tibial angle (MPTA), mechanical lateral distal femoral angle (mLDFA), and mechanical axis deviation (MAD) on anteroposterior radiographs encompassing the entire lower extremity during the preoperative and postoperative first year. In order to determine the degree of arthritis, The Kellgren-Lawrence (KL) grading system was employed on preoperative and the most recent anteroposterior knee radiographs of the patients. MME, the distance (in millimeters) between the peripheral border of the meniscus body (meniscocapsular junction) and the medial border of the tibial plateau, was measured and calculated on coronal MRI. Preoperative and postoperative measurements of MPTA, MAD, MME, and KL staging were compared.

Results

The study included a total of 21 patients, comprising 7 males and 14 females. Among these, 6 were left-sided and 15 were right-sided cases, with an average age of 52.2 (±6.1) years. The mean follow-up duration for the patients was 5.4 (±2.3) years, with an average time of 2.2 (±1.6) years from surgery to the MRI. While significant differences were observed between preoperative and postoperative measurements for MAD and MPTA (p <0.01), no significant difference was found in MME measurement (p: 0.507).

Pearson correlation analysis was employed to determine the correlation between preoperative and postoperative values of MME, MPTA, and MAD. A significant negative correlation was observed between preoperative MME and MPTA (r: -0.464, p:0.034). No significant correlation was found between postoperative MME and MAD or MPTA. Comparisons based on KL staging between the preoperative and postoperative periods did not reveal any significant differences (p: 0.525).

Conclusion

In knees with both MMPRT and varus alignment, our study demonstrated that postoperative MME and radiological progression of arthritis did not increase after HTO without MMPRT repair. These findings suggest that HTO treatment performed without MMPRT repair may prevent an increase in MME and the progression of arthritis. According to the results of our study, we observed a negative correlation between MME and MPTA during the preoperative period, which supports the relationship between varus deformity and MME.

## Introduction

Medial meniscus posterior root tear (MMPRT) is defined as a complete full-thickness radial tear occurring within 1 cm of the tibial attachment site of the posterior meniscus [1,2]. During weight-bearing, the load in the knee is distributed by the meniscus. However, in the case of an MMPRT, the knee's biomechanics are disrupted, leading to an increase in contact pressure [3]. Biomechanical studies have demonstrated that there is no significant difference in tibial contact pressure increase between MMPRT and total meniscectomy [3].

A typical finding of a medial meniscal extrusion (MME) root tear, it is considered as one of the significant factors affecting the disruption of load distribution and the acceleration of joint degeneration [1,4]. The impact of MMPRT repair on meniscal extrusion is still a subject of debate [5]. High tibial osteotomy (HTO) has been an effective treatment method for patients with single-compartment knee osteoarthritis associated with varus deformity for many years [6,7]. While still controversial, varus deformity is considered a risk factor for MMPRT [8]. There is no consensus in the literature regarding the treatment method for patients with MMPRT and varus alignment of the knee. Despite opposing views, there are numerous publications indicating that clinical outcomes are favorable even though the healing rates for MMPRT following HTO may be low [5,9,10]. In these publications, it is emphasized that correcting the alignment of the knee is more important. Additionally, it has been demonstrated that MME can persist even after HTO, independent of clinical outcomes [5,11]. The relationship between MME and clinical outcomes after HTO is not entirely clear or well-established.

Our hypothesis is that in patients with MMPRT and varus deformity of the knee, without root repair, MME will persist after HTO and radiological staging will worsen. The objective of this study is to compare the preoperative and postoperative radiological outcomes, with a particular emphasis on MME, in patients who undergo HTO without MMPRT repair.

## Materials and methods

After obtaining ethical committee approval (Protocol code: SÜKAEK-2023 16/6), patients diagnosed with medial compartment osteoarthritis who underwent open wedge HTO between January 2015 and December 2020 were retrospectively reviewed. The inclusion criteria were defined as patients who had preoperative and postoperative magnetic resonance imaging (MRI) and weight-bearing radiographs encompassing the entire lower extremity. Following data screening, patients in whom LaPrade Type 2 MMPRT was detected on preoperative MRI were included in the study [12]. Patients who underwent meniscal repair or meniscectomy, those who developed deformity after trauma, those with ligament injuries, and those with a history of knee fractures were excluded from the study. Demographic data such as the age and gender of the patients were retrospectively recorded.

Radiological evaluation

**Figure 1:**
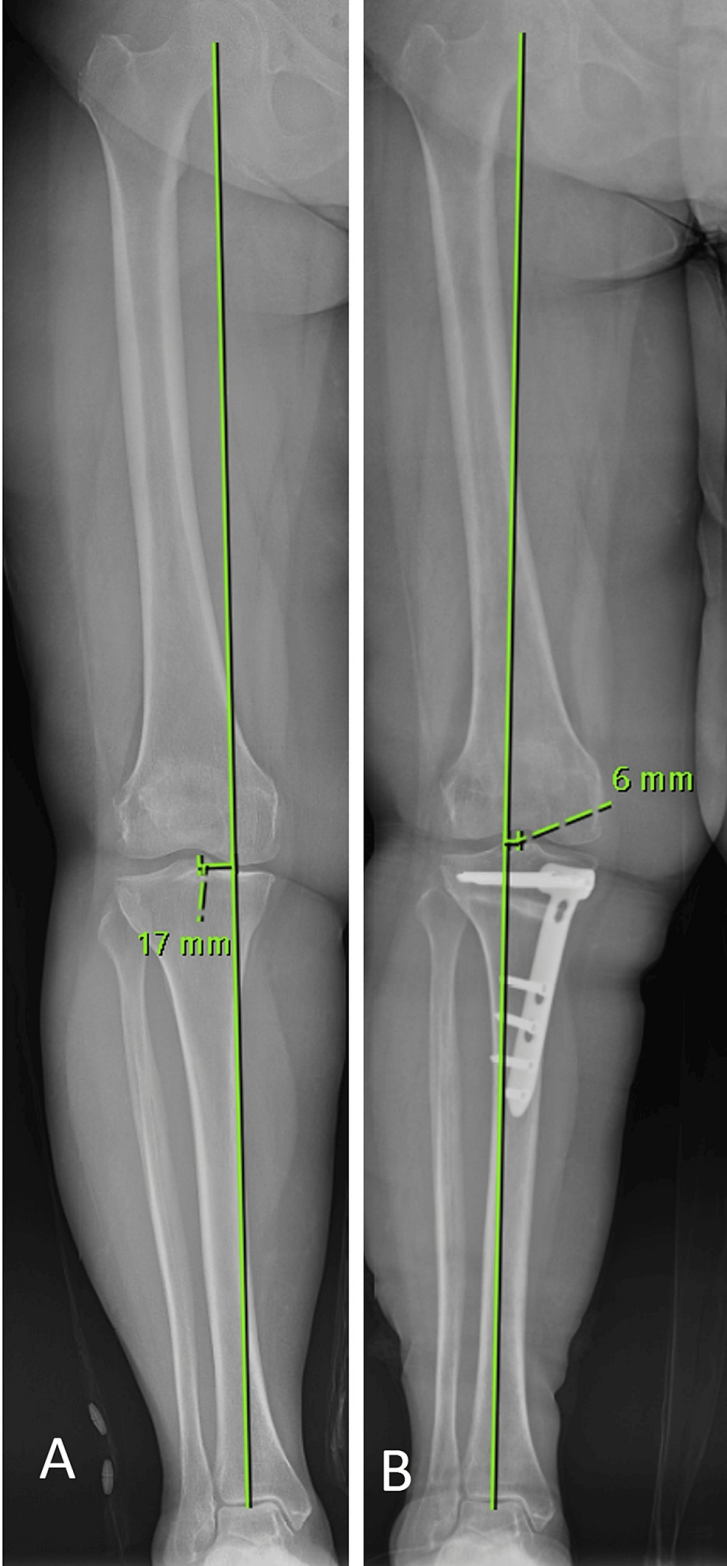
Displaying radiological measurements of WBL and MAD. In anteroposterior radiographs encompassing the entire lower extremity, the line drawn from the center of the femoral head to the midpoint of the superior talar joint line is used to obtain WBL. A: In the preoperative image, WBL passes through the medial joint space. B: In the postoperative image, it is observed that WBL deviates laterally from the center of the knee joint. WBL: Weight-bearing line; MAD: Mechanical axis deviation.

**Figure 2:**
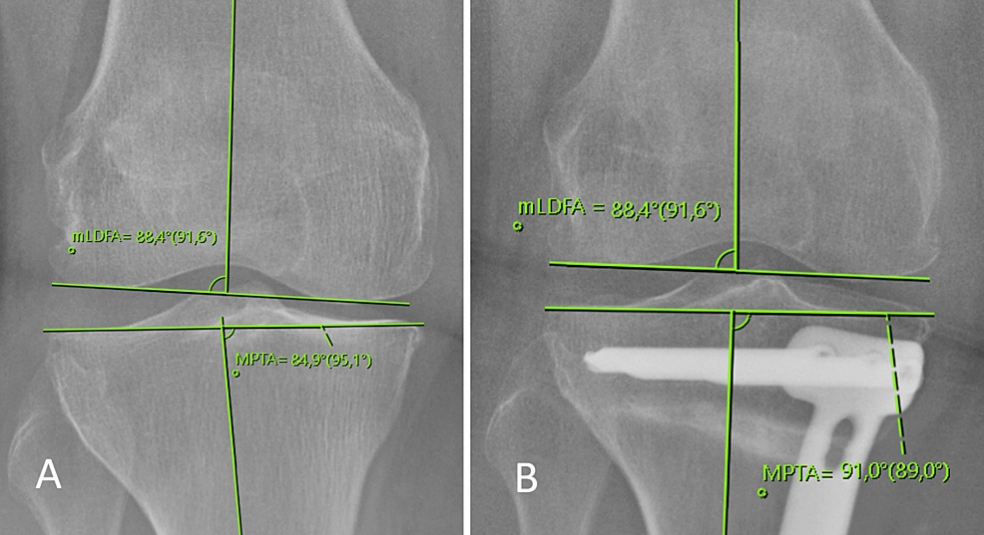
Displaying radiological measurements of mLDFA and MPTA. A: Display of preoperative MPTA (84.9°) and mLDFA (88.4°) measurements. B: Display of postoperative MPTA (91°) and mLDFA (88.4°) measurements. mLDFA: Mechanical lateral distal femoral angle; MPTA: Medial proximal tibial angle.

**Figure 3:**
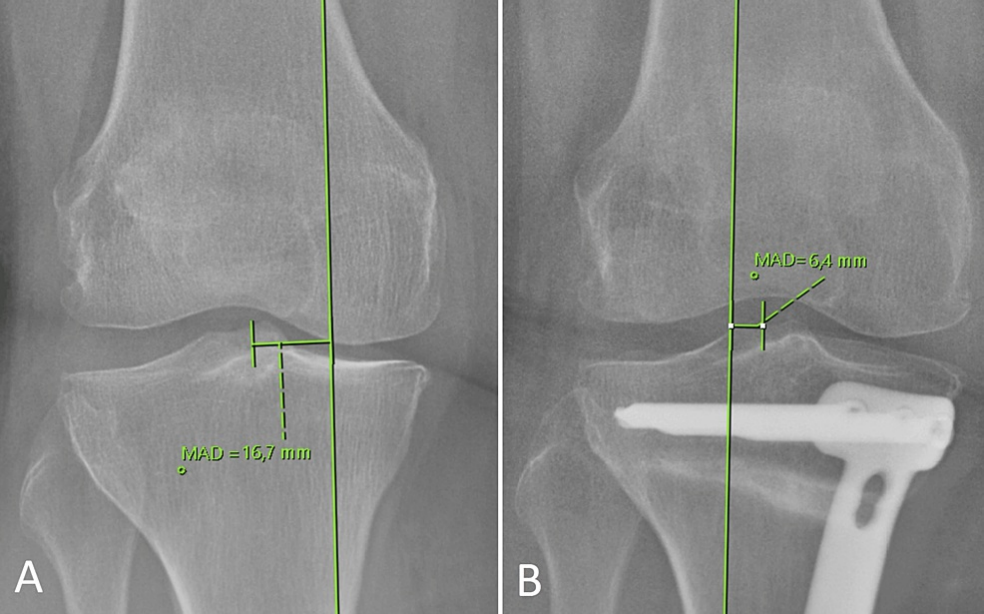
Measuring MAD based on the deviation from the joint center of WBL. A: Preoperative MAD is calculated as 16.7 mm. B: Postoperative MAD is calculated as a negative value (-6.4 mm) because it is located lateral to the center of the knee joint. WBL: Weight-bearing line; MAD: Mechanical axis deviation.

The measurements of medial proximal tibial angle (MPTA), mechanical lateral distal femoral angle (mLDFA), and mechanical axis deviation (MAD) were performed on preoperative and postoperative anteroposterior radiographs encompassing the entire lower extremity of the patients, as described in the literature [13]. Weight-bearing line (WBL), to evaluate the mechanical axis of the lower extremity in the coronal plane, was obtained by drawing a line from the center of the femoral head to the midpoint of the superior articular surface of the talus (Figure 1). MPTA was calculated as the angle formed medially between the proximal tibial joint orientation line and the mechanical axis of the tibia (Figure 2). mLDFA was calculated as the angle formed laterally between the distal femoral joint orientation line and the mechanical axis of the femur (Figure 2). MAD was calculated as the distance of WBL from the center of the knee joint (Figure 3). WBL, knee joint center being on the medial side was recorded as a positive value while being on the lateral side was recorded as a negative value.

**Figure 4:**
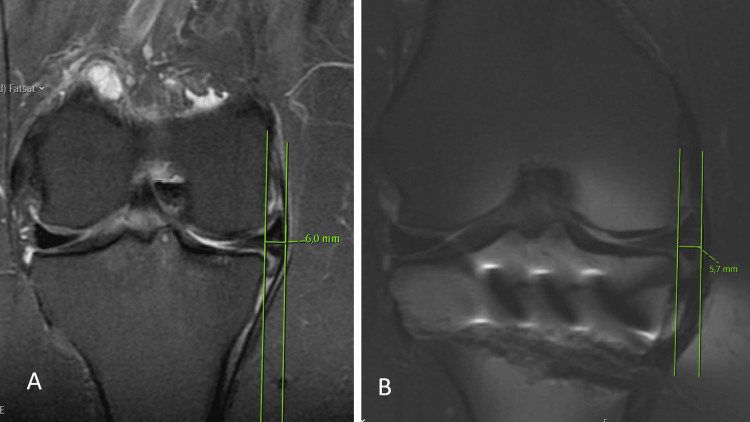
Displaying preoperative and postoperative MME measurements. MME, as measured on coronal section MRI, is the distance between two parallel lines drawn from the medial border of the tibial plateau to the periphery border of the meniscus body. A and B: In the same patient, the preoperative MME was measured as 6 mm (A), and the postoperative MME was measured as 5.7 mm (B). MME: Medial meniscal extrusion; MRI: Magnetic resonance imaging.

In the preoperative and most recent anteroposterior knee radiographs obtained, the KL grading system was used to determine the degree of arthritis (grade 1 indicates doubtful joint narrowing; grade 2 indicates minimal joint narrowing and distinct osteophytes; grade 3 indicates marked joint narrowing, sclerosis, and multiple osteophytes; grade 4 indicates severe joint space narrowing, severe subchondral sclerosis, osteophytes, and subchondral cysts) [14]. MME was calculated by measuring the distance (in millimeters) on coronal MRI between the peripheral border of the meniscus body (meniscocapsular junction) and the medial border of the tibial plateau (Figure 4). The coronal MRI section was selected so that it was precisely in the middle of the medial tibial plateau in the sagittal plane [15].

Surgical technique and postoperative rehabilitation

Initially, all patients underwent diagnostic arthroscopy. HTO was performed if, during diagnostic arthroscopy, there were grade 2 or lower lesions in the lateral articular cartilage according to the International Cartilage Relief Society (ICRS) classification. A longitudinal incision of approximately 4 cm was made on the proximal anteromedial tibia. After partially releasing the pes anserinus and superficial fibers of the medial collateral ligament (MCL), two guide pins were directed along the planned osteotomy line. Following that, a biplanar open wedge osteotomy was performed with the assistance of a saw and osteotome. With the assistance of a posteriorly placed separator, the osteotomy line was gradually brought to the planned opening. In its final position, the osteotomy line was fixed with a locking plate.

Patients were not allowed to bear weight for the first two weeks. Partial weight-bearing was initiated at the end of the second week. If tolerated by the patients, full weight-bearing was allowed at the end of the first month. Passive exercises were initiated starting from postoperative day one to achieve a joint range of motion, and isometric exercises were started at the end of the first week.

Statistical analysis

Statistical analysis was performed using the Statistical Package for Social Sciences (SPSS) software version 21 (IBM Corp, Armonk, NY, USA). Categorical variables were presented as frequencies and percentages, while continuous numerical variables were presented as medians. Continuous variables were examined for homogeneity of variances, and a normal distribution analysis was conducted. The chi-square test was employed to compare frequencies. A paired sample T-test was employed to compare measurements taken before and after surgery, including MAD, MPTA, and MME. The correlation between MME and MAD, as well as MPTA, was analyzed using the Pearson correlation test. A p-value below 0.005 was considered statistically significant.

The measurements on MRI were performed by two experts (YSK and HA) with more than 10 years of orthopedic experience. All measurements were independently conducted in a blinded manner, with no knowledge of each other's results. To assess interobserver agreement, additional measurements were performed on 20 randomly selected MRI images among the observers. Interclass correlation coefficients (ICCs) were calculated based on the measurements. The ICC values were calculated as follows: 0.869 for preoperative MAD, 0.821 for preoperative MPTA, 0.953 for preoperative MME, 0.933 for postoperative MAD, 0.861 for postoperative MPTA, and 0.953 for postoperative MME. The interobserver reliability was found to be satisfactory.

## Results

A total of 21 patients were included in the study, comprising 7 males and 14 females. It was observed that HTO was applied to the left side in 6 patients and to the right side in 15 patients. The average age of the patients was 52.2 (±6.1) years. The average follow-up duration for the patients was 5.4 (±2.3) years, with an average time of 2.2 (±1.6) years between surgery and the MRI examination. The radiological measurement results of the patients before and after surgery were as follows: (1) MAD: Before surgery: 22.07 mm (±8.3), after surgery: -2.76 mm (±6.1); (2) MPTA: Before surgery: 84.60 (±1.9), after surgery: 91.40 (±1.9); and (3) MME: Before surgery: 5.02 mm (±1.2), after surgery: 4.91 mm (±1.1) (Table 1).

**Table 1:** The means of the quantitative data in the study. A negative (-) value indicates lateralization of MAD, N: Total number of cases. Preop: Preoperative; Postop: Postoperative; MAD: Mechanical axis deviation; MPTA: Medial proximal tibial angle; LDFA: Lateral distal femoral angle; MME: Medial meniscus extrusion; mm: Millimeter; °: Degree.

	N	Mean	Standard Deviation
Age (years)	21	52.2	6.1
Preop MAD(mm)	21	22.07	8.3
Postop MAD (mm)	21	-2.76	6.1
Preop MPTA (°)	21	84.6	1.9
Postop MPTA (°)	21	91.4	1.9
Preop MME (mm)	21	5.02	1.2
Postop MME (mm)	21	4.91	1.1
Follow-up duration (years)	21	5.4	2.3
LDFA (°)	21	89	1.6
Postoperative MRI interval (years)	21	2.2	1.6

There was a significant difference observed in the measurements of MAD and MPTA between preoperative and postoperative periods (p < 0.01), while there was no significant difference detected in MME measurements (p: 0.507) (Table 2). The mean mLDFA for patients was measured at 89 degrees.

**Table 2:** Comparison of radiological measurements before and after surgery. A dependent samples T-test was used to compare preoperative and postoperative data. A statistical significance level of p<0.05 was considered. Preop: Preoperative; Postop: Postoperative; MAD: Mechanical axis deviation; MPTA: Medial proximal tibial angle; MME: Medial meniscus extrusion; mm: Millimeter; °: Degree. In the comparison conducted between preoperative and postoperative measurements, significant differences were observed in terms of MPTA and MAD (P<0.05), while no significant difference was found in terms of MME (p:0.507).

	Difference Between the Means	Standard Deviation	95% Confidence Interval		P-value
			Lower	Upper	
Preop MAD - Postop MAD (mm)	24.8	8.8	20.8	28.8	0.00
Preop MPTA - Postop MPTA (°)	6.8	2.8	5.5	8.06	0.00
Preop MME - Postop MME (mm)	0.11	0.77	-0.23	0.46	0.507

Pearson correlation analysis was used to determine the correlation between preoperative and postoperative MME, MPTA, and MAD values. All data are summarized in Table 3. A significant negative correlation was observed between preoperative MME and MPTA (r: -0.464, p:0.034). No significant relationship was found between postoperative MME and MAD or MPTA.

**Table 3:** Evaluation of preoperative and postoperative correlation between radiological measurements. Pearson correlation test was used to conduct correlation analysis. *The correlation is significant at the p<0.05 level. The correlation coefficient was defined as weak (0.01-0.29), moderate (0.30-0.70), and strong (0.71-0.99) in terms of strength. Pearson r: Correlation coefficient; Preop: Preoperative; Postop: Postoperative; MAD: Mechanical axis deviation; MPTA: Medial proximal tibial Angle; MME: Medial meniscus extrusion. There was a moderate negative correlation (-0.464) found between preop MPTA and preop MME, which was statistically significant (p: 0.034).

		Preop MAD	Preop MPTA
Preop MME	Pearson r	0.240	-0.464^*^
	p-value	0.295	0.034
		Postop MAD	Postop MPTA
Postop MME	Pearson r	0.191	-0.101
	p-values	0.408	0.663

**Figure 5:**
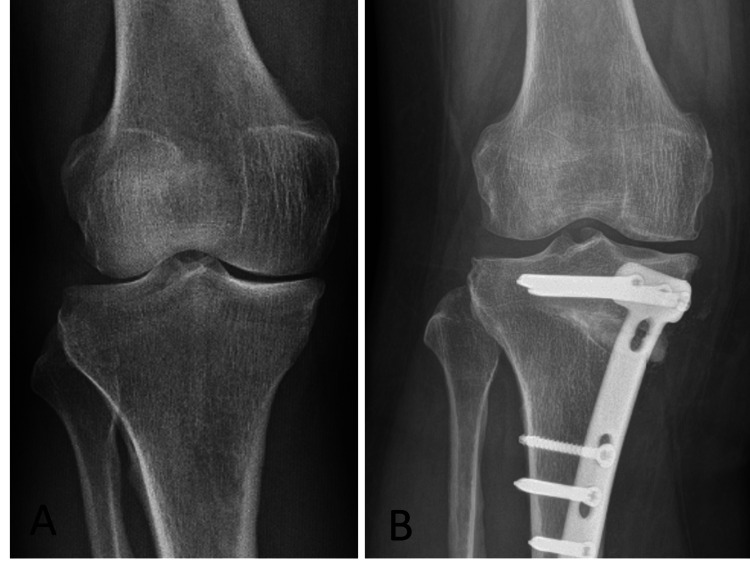
Evaluation of Kellgren-Lawrence grading in preoperative and postoperative radiographs. A patient has been shown to regress from Kellgren-Lawrence grade 3 (A) to grade 2 (B) postoperatively.

In the preoperative period, according to the KL grading system, grade 2 arthritis was detected in 14 patients, while grade 3 arthritis was detected in seven patients During follow-up, it was observed that three patients who had preoperative grade 2 arthritis progressed to grade 3, while one patient regressed from preoperative grade 3 to grade 2 (Figure 5). 

In the comparison based on the KL grading between the preoperative and postoperative periods, no significant difference was observed (p: 0.525) (Table 4).

**Table 4:** Comparison of preoperative and postoperative medial compartment osteoarthritis. Chi-squared test was used to compare preoperative and postoperative data. A statistical significance level of p<0.05 was considered, n: Number of cases. Preop: Preoperative; Postop: Postoperative; MCOA: Medial compartment osteoarthritis KL: Kellgren-Lawrence. No significant change was observed in patients' preoperative and postoperative arthrosis conditions based on the KL grading (P:525).

		KL grading		Total (n)	P value
		Grade 2 (n)	Grade 3 (n)		
Surgical Status	Preop (n)	14	7	21	0.525
	Postop (n)	12	9	21	

## Discussion

The most significant finding of this study is that in patients with varus knee deformity accompanied by MMPRT, there was no significant change in MME following HTO without concomitant meniscectomy or meniscus repair, compared to the preoperative period. Furthermore, it was observed that there was no significant progression according to the KL grading in radiological follow-ups over an average of 5 years in the postoperative period of the patients.

MMPRT has been biomechanically shown to be equivalent to total meniscectomy, leading to a disruption in tibiofemoral load distribution and an increase in contact pressure, ultimately resulting in the development of early chondral damage [1-3]. Biomechanical studies have demonstrated that after MMPRT repair, contact pressure and load distribution approach normal levels [16]. Therefore, if advanced-stage osteoarthritis is not present, MMPRT repair is preferred as a primary treatment option. In knees with concomitant MMPRT and varus deformity, there is no consensus in the literature regarding treatment options. Many studies indicate that patients who undergo HTO alone without addressing the root tear achieve good clinical and radiological outcomes, even without healing of the root tear [9,11,17-19]. Recently, there have been opposing views to this perspective. Suh et al. emphasized in their study that the results of HTO accompanied by MMPRT repair are more effective [20]​​​​​​. In our study, it was observed that patients who underwent only HTO without MMPRT repair did not show an increase in arthritis according to the KL grading after an average follow-up of 5 years. This finding can be interpreted as HTO application without root repair being able to prevent the progression of arthritis in patients with MMPRT, and this result is supported by the literature.

MME, known as the displacement of the meniscus body from the edge of the tibial plateau, is one of the radiological diagnostic criteria for MMPRT. Ideally, MMPRT healing should prevent the progression of MME. However, many biomechanical and clinical studies have shown that in MMPRTs, there is a significant increase in MME even after repair, but this condition is not associated with poor clinical outcomes. In their study, Moon et al. reported an increase in MME only after MMPRT repair without HTO [21]. In a study conducted by Ke et al., it was noted that even in patients with complete healing of the meniscal root, MME did not show improvement [18]. The reason why MME continues to persist even after meniscal healing is not yet fully understood. Furthermore, studies have also indicated that in varus knees accompanied by MMPRT, MME does not decrease after HTO [22]. In a study by Lee et al., it was emphasized that the postoperative meniscal extrusion did not change compared to the preoperative period in both patients who underwent MMPRT repair and those who did not after HTO [17]. In our study, there was no significant change in postoperative MME, which is consistent with the existing literature. In a study by Furumatsu et al., they evaluated the progression of MME after acute MMPRT [23]. They found that MME progressed gradually, with an average increase of 3 mm in the first month, 4.2 mm by the third month, and up to 5.8 mm between 4 to 12 months. In a study by Krych et al., they suggested that meniscotibial ligament abnormalities exist in meniscal extrusions of more than 3 mm, and therefore, meniscal centralization may be beneficial in reducing the amount of MME [24]. In our study, the absence of meniscal centralization may explain the lack of change in postoperative MME quantity. However, future studies may demonstrate that early HTO treatment or meniscal centralization performed before MME progression based on symptoms can lead to a postoperative reduction in MME quantity. Further studies are needed to elucidate this matter.

Varus alignment is considered a poor prognostic factor for root repair [21]. An increase in MME quantity after root repair is a risk factor for poor meniscal healing. In a biomechanical study conducted by Willinger et al., it was shown that MME quantity and contact pressure were greater in varus-aligned knees compared to valgus and neutral alignment [25]. In a study by Jing et al., it was stated that having the mechanical axis in varus increased the risk of MMPRT by 3.3 times and that MME was associated with varus alignment [19]. In the literature, it is generally accepted that in knees with MMPRT accompanied by varus deformity, correction of lower extremity alignment should be the primary consideration. In our study, postoperative MAD lateralization and a postoperative mean MPTA above 90 degrees indicate deviation of the lower extremity mechanical axis from medial to lateral. Furthermore, in our study, a negative correlation was found between preoperative MME and MPTA. These findings support the relationship between varus deformity and MME in our study, which is consistent with the literature.

This study has natural limitations due to its retrospective nature and a small sample size. It is designed as a radiological study and does not reflect clinical outcomes. Additionally, the absence of a control group with root repair and the lack of evaluation of arthritis with diagnostic arthroscopy in the postoperative period are other significant limitations.

## Conclusions

In patients who underwent only HTO without MMPRT repair, no increase in arthritis according to KL staging was observed, and furthermore, a significant change in postoperative MME quantity compared to the preoperative period was not detected. These findings suggest that HTO treatment performed without MMPRT repair may prevent an increase in MME and the progression of arthritis. According to the results of our study, we observed a negative correlation between MME and MPTA during the preoperative period, which supports the relationship between varus deformity and MME and strengthens the existing scientific evidence in the literature.
